# Supplementary material for: Identification of Freezing-Responsive microRNAs and Their Targets in Chinese Jujube by Small RNA and Degradome Sequencing
Source: Int J Mol Sci. 2026 Sep 4;27(17):7912. doi: 10.3390/ijms27177912 (PMC13566890; doi:10.3390/ijms27177912)
Supplement: Supplementary file 1 [file ijms-27-07912-s001.zip › Supplementary Material S2/novel_miR_64.pdf]

Provisional ID	:	novel_miR_64
Score total	:	1.8
Score for star read(s)	:	-1.3
Score for read counts	:	0
Score for mfe	:	1.5
Score for randfold	:	1.6
Score for cons. seed	:	
Total read count	:	361
Mature read count	:	215
Loop read count	:	146
Star read count	:	0

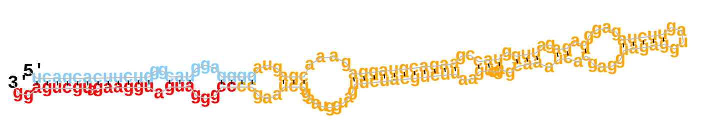

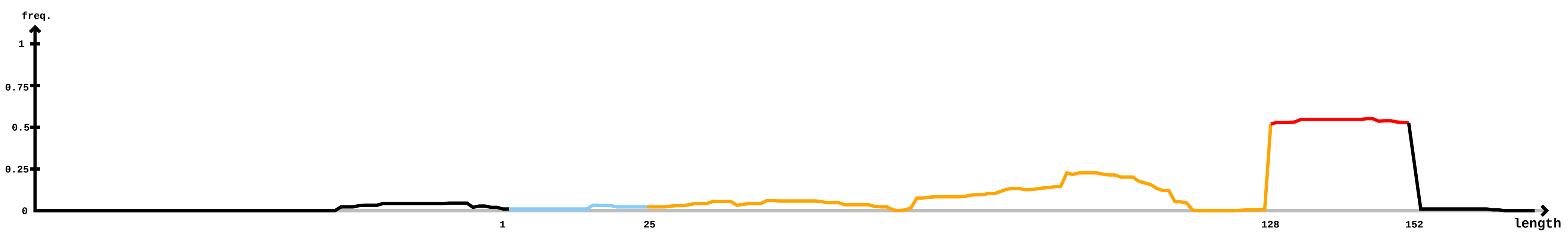

Star Mature

```
5'- cagaauuaugcaaggggguuucagcuucuugcugagccuggaagaggguuaauaacagccggaagccugagaaagaauucagcacuucugggcauggaggggaugagcaaagaggaugcagaagccaugguuagagagggagaucuugauggagauggagcacuaaacgagacugaauucugcaucuugaugguaaggcuaagcccccgggaugauggaagaugcugaggcauggcuugagaaggcuauuga -3'
```

exp	reads	mm	sample
aauCacagccggaagccugaga	1	1	JT-1
aauaacagccggaagccugaga	8	0	JT-1
aacagccggaagccugagaaag	3	0	LCK-3
acagccggaagccugaga	1	0	LT-3
gccggaagccugagaaagaa	2	0	JT-3
gccggaagccugagaaagaa	2	0	JT-2
gagaaagaauucagcacuucugggca	1	0	LT-2
agaauucagcacuucugggcaug	1	0	JCK-3
agaauucagcacuucugggcaug	1	0	JCK-1
agaauucagcacuucugggcaug	1	0	JCK-2
cauggaggggaugagcaaagagga	5	0	JT-2
cauggaggggaugagcaaagagga	4	0	JT-3
agcaaagaggaugcagaa	1	0	LT-1
agcaaagaggaugcagaagccaugg	1	0	LT-3
gcaaagaggaugcagaagccauggu	1	0	LT-3
aaagaggaugcagaagccauggu	2	0	LT-2
aagaggaugcagaagccaugguuagagagg	3	0	JCK-3
aggaugcagaagccaugguuag	5	0	LCK-3
gcagaagccaugguuagagagggaga	2	0	LCK-3
cagaagccaugguuagagagg	1	0	LT-3
cagaagccaugguuagagaggg	1	0	LT-1
aagccaugguuagagagggag	7	0	LCK-3
cuugauggagauggagcacuaaac	1	0	LT-2
cuugauggagauggagcacuaaacga	1	0	LT-2
uugauggagauggagcacuaaacgaga	2	0	JT-2
uugauggagauggagcacuaaacgaga	2	0	JT-3
ugauggagauggagUacu	1	1	JCK-2
ugauggagauggagUacu	1	1	LT-1
ugauggagauggagUacu	1	1	JCK-3
ugauggagauggagUacu	16	1	JT-1
ugauggagauggagUacu	3	1	LT-3
ugauggagauggagcacuaaa	1	0	LT-2
ugauggagauggagcacuaaacg	1	0	LT-1
auggagauggagcacuaaacgag	1	0	LT-1

Star Mature

cagaauuaugcaaggggguuucagcuucuugcugagccuggaagaggguuaauaacagccggaagccugagaaagaauucagcacuucugggcauggaggggaugagcaaagaggaugcagaagccaugguuagagagggagaucuugauggagauggagcacuaaacgagacugaauucugcaucuugaugguaaggcuaagccccgggaugauggaagaugcugaggcauggcuugagaaggcuauuga

auggagauggagcacuaaacgagacugaUu	1	1	JCK-2
uggagauggagcacuaaacgaga	1	0	LT-3
auggagcacuaaacgagacugaau	1	0	LT-3
uggagcacuaaacgagacugaa	1	0	LT-2
uggagcacuaaacgagacugaa	2	0	LCK-3
ggagcacuaaacgagacugaauuc	1	0	LT-1
agcacuaaacgagacugaauuc	1	0	LT-2
agNacuaaacgagacugaauuc	1	1	LT-2
agcacuaaacgagacugaauuc	1	0	LT-1
cacuaaacgagacugaauucugc	5	0	JT-1
acuaaacgagacugaauucugc	1	0	LT-1
acuaaacgagacugaauucugca	3	0	LT-3
acuaaacgagacugaauucugca	1	0	LT-1
cuaaacgagacugaauuc	1	0	LT-3
cuCaacgagacugaauucugc	1	1	JCK-2
aaacgagacugaauucugc	3	0	LCK-3
aaacgagacugaauucugcau	4	0	JCK-1
aaacgagacugaauucugcauc	2	0	LT-3
aaacgagacugaauucugcauc	5	0	LT-2
aaacgagacugaauucugcaucu	5	0	LCK-3
acgagaGugaauucugcaucuug	2	1	LT-3
cgagacugaauucugcauc	1	0	LT-2
cgagacugaauucugcaucuug	1	0	LT-3
cgagaGugaauucugcaucuugau	1	1	LT-1
gagaGugaauucugcauc	1	1	LT-1
gagaGugaauucugcaucuug	1	1	LT-3
agaGugaauucugcaucuugaug	1	1	LT-1
agacugaauucugcaucuugaug	1	0	LT-3
agaGugaauucugcaucuugaug	1	1	LT-3
gaGugaauucugcaucuug	2	1	LT-3
acugaauucugcaucuug	20	0	JT-1
acugaauucugcaucuuga	1	0	LT-2
aGugaauucugcaucuugau	2	1	LT-1
aGugaauucugcaucuugaug	9	1	LCK-3
aGugaauucugcaucuugaugg	1	1	LT-3
cugaauucugcaucuugaug	1	0	LT-3
ugaauucugcaucuugaug	4	0	LCK-3
aagccccgggaugauggaagaug	1	0	LT-1
agccccgggaugauggaagaug	1	0	LT-3
cccgggaugauggaagaugcugagg	1	0	LT-1
ccgggaugauggaagaug	4	0	LCK-3
ccgggaugauggaagaugcug	1	0	LT-3
ccgggaugauggaagaugcugag	1	0	LT-3
ccgggaugauggaagaugcugagg	31	0	LCK-2
ccgggCugauggaagaugcugagg	1	1	LCK-2
ccgggaugCuggaagaugcugagg	1	1	LCK-3
ccAggaugauggaagaugcugagg	1	1	LT-2
ccgggaugauggaagaugcugagg	58	0	LCK-3
ccgggaugauggaagaugcugagg	23	0	LT-2
ccgggaugauggaagaugcugagg	18	0	LT-3
ccgggaugauggaagaugcugagg	34	0	LCK-1
ccgggaugauggaagaugcugagg	4	0	JT-3
ccgggaugauggaagaugcugagg	24	0	LT-1
ccgggaugauggaagaugcugagg	2	0	JT-2
cgggaugauggaagaugc	1	0	LT-2
cgggaugauggaagaugcug	2	0	LT-1
cgggaugauggaagaugcuga	1	0	JCK-3
gaugauggaagaugcugaggc	1	0	LT-2
augauggaagaugcugaggc	6	0	LCK-3
ugcugaggcauggcuugagaa	2	0	LT-2
ugaggcauggcuugagaagg	2	0	LT-1
